# Treatment of non-odontogenic orofacial pain using botulinum toxin-A: a retrospective case series study

**DOI:** 10.1186/s40902-018-0159-z

**Published:** 2018-08-06

**Authors:** Sang-Yun Kim, Young-Kyun Kim, Pil-Young Yun, Ji-Hyun Bae

**Affiliations:** 1 Department of Oral and Maxillofacial Surgery, Section of Dentistry, Seoul National University Bundang Hospital, Seongnam, South Korea; 2 Department of Dentistry and Dental Research Institute, School of Dentistry, Seoul National University, Seoul, South Korea; 3 Department of Conservative Dentistry, Section of Dentistry, Seoul National University Bundang Hospital, 300 Gumi-dong, Bundang-gu, Seongnam city, Gyunggi-do South Korea

**Keywords:** Orofacial pain, Botulinum toxin, BTX-A

## Abstract

**Background:**

The purpose of this study was to evaluate the clinical outcomes of treatment of non-odontogenic atypical orofacial pain using botulinum toxin-A.

**Methods:**

This study involved seven patients (seven females, mean age 65.1 years) who had non-odontogenic orofacial pain (neuropathic pain and atypical orofacial pain) and visited the Seoul National University Bundang Hospital between 2015 and 2017. All medication therapies were preceded by botulinum toxin-A injections, followed by injections in the insignificant effects of medication therapies. Five of the seven patients received intraoral injections in the gingival vestibule or mucosa, while the remaining two received extraoral injections in the masseter and temporal muscle areas.

**Results:**

In five of the seven patients, pain after botulinum toxin-A injection was significantly reduced. Most of the patients who underwent surgery for dental implantation or facial nerve reconstruction recovered after injections. However, the pain did not disappear in two patients who reported experiencing persistent pain without any cause.

**Conclusions:**

The use of botulinum toxin-A for the treatment of non-odontogenic neuropathic orofacial pain is clinically useful. It is more effective to administer botulinum toxin-A in combination with other medications and physical therapy to improve pain.

**Electronic supplementary material:**

The online version of this article (10.1186/s40902-018-0159-z) contains supplementary material, which is available to authorized users.

## Introduction

When pain occurs in the orofacial area, it is very important to identify and diagnose its cause. Orofacial pain can be classified into temporomandibular joint disorders, muscle disorders, angiogenic pain, neuropathic pain, cardiogenic pain, and atypical facial pain [1].

Tissue or nerve damage causes inflammation, and the initial inflammatory response plays a very important role in the onset of neuropathic pain. Especially, the structures of the trigeminal nervous system are placed in a closed space, so that the pressure generation due to inflammation can cause nerve damage. After the apparent trauma experience such as dental surgery such as implant, extraction, orthognathic surgery, endodontic treatment, and fracture, it continues to suffer from burning pain. The neuropathic pain, which is presumed to be caused by trauma, is called painful traumatic trigeminal neuropathy and is distinguished from classical trigeminal neuralgia [1, 2].

Atypical facial pain may be defined as a diffuse pain deep within the tissue, which persists without an obvious disease or cause. Pain patterns range from dull, tingling, to sharp or throbbing pain. The duration of the pain may range from intermittent to persistent. This type of pain is difficult to treat because symptoms vary and its cause is unclear. In some cases, medications such as analgesics, tricyclic antidepressants, and anticonvulsants are effective, but in many cases, medication alone is not sufficient for treatment. Recently, treatment with botulinum toxin-A has been introduced [2].

Botulinum toxin is a type of neurotoxin produced by *Clostridium botulinum*. It plays a role in suppressing extracellular release of synaptic vesicles by acting on the nerve endings at the neuron junctions of mammals. It also affects autonomic nerves by inhibiting the release of acetylcholine from nerve junctions in the sweat glands and smooth muscles. Based on this principle, botulinum toxin-A can be applied for the treatment of neuromuscular diseases, especially focal tension disorders or seizures. Although botulinum toxin-A is very effective for direct muscle relaxation, it may also modulate pain through peripheral nociceptors by inhibiting the exocytosis of various neurotransmitters [3, 4]. The purpose of this study was to analyze cases retrospectively and evaluate the clinical efficacy of botulinum toxin-A in patients with non-odontogenic orofacial pain.

## Review

Among the patients who visited the dental clinic of Seoul National University Bundang Hospital between 2015 and 2017, we studied seven patients who complained of non-odontogenic orofacial pain. Five patients were diagnosed with painful traumatic trigeminal neuropathy and two others were diagnosed with atypical orofacial pain. This study was conducted with the approval of the Institutional Review Board of the Seoul National University Bundang Hospital (IRB No. B-1802-453-107). All patients were treated with medication and physical therapy. However, these methods were either ineffective or had minimal effect. Botulinum toxin-A (BTX-A) injection therapy was therefore performed. The age, sex distribution, diagnosis, and treatment methods and course of patients were summarized using their medical records (Table 1).

**Table 1:** Patients’ information

No.	Age	Sex	Area	Primary medication	Physical therapy	Type of BTX-A	Dose of BTX-A (U)	Numbers of BTX-A injection	Effect	Injection sites	Complications
1	70	F	Right upper anterior gingiva	Tegretol, Trileptal, Neurontin, Lyrica	Soft laser	Meditoxin	120	1	X	Intraoral	No
2	67	F	Left upper lip	Tegretol, Amitriptyline, Trileptal, Neurontin	EAST	Dysport	200	2	O	Intraoral	Unnatural lip movement
3	62	F	Right lower lip, chin	Trileptal, Sensival, Lyrica, Neurontin	EAST, soft laser	Meditoxin	100	2	O	Intraoral	Unnatural lip movement
4	52	F	Left face	Trileptal, Neurontin, Sensival	Soft laser	Innotox	25	3	O	Extraoral	No
5	71	F	Left face, lip, gingiva	Trileptal, Neurontin	Soft laser	Innotox	20	1	O	Intraoral	Transient facial unbalance
6	68	F	Both face	No	Soft laser	Dysport	250	1	O	Extraoral	No
7	66	F	Left upper posterior gingiva	Trileptal, Neurontin, Sensival	Soft laser	Innotox	20	1	X	Intraoral	Unnatural lip movement

*X* non-effective, *O* effective

### Case 1 (Table 1, no. 2)

A 67-year-old woman visited our hospital. She complained of severe pain in the maxillary left anterior buccal vestibule. She also reported that her upper lip has tightened after multiple dental implants were placed in her maxilla. Clinically and radiologically, painful traumatic trigeminal neuropathy was diagnosed. Two hundred milligrams of Tegretol® (Novartis, Basel, Switzerland) was administered twice a day for 2 weeks, and the symptoms were alleviated, but the tightened pain on the bilateral sides of the nose persisted. The dose was therefore increased for another 2 weeks. Most of the pain was alleviated, but the pain that occurred when she moved her mouth was still present. She therefore received the medication for two additional weeks. The frequency and severity of the pain was reduced, but side effects such as headache and dizziness occurred; therefore, 10 mg of amitriptyline (Myung-In, Seoul, Korea) was administered once a day before bedtime and capsaicin ointment (Dipental cream, Dalim BioTech, Seoul, Korea) was applied locally at the site of the pain. A stent was made in the maxilla and capsaicin ointment was applied to the stent for 20 min. This stent was to be worn three times a day. However, no improvement was observed and the pain worsened. Thus, 100 mg of Neurontin (Pfizer Ltd., Seoul, Korea) was administered three times daily. Electric acupuncture stimulation therapy (EAST, Pulse Generator (PG)-8® (ITO Co., Tokyo, Japan)) was performed four times at 2-week intervals, but the burning and throbbing pain on the upper lip and tenderness of the palatal area remained. The medication of the patient was changed to 300 mg of Trileptal (Novartis, Basel, Switzerland) which was administered twice daily. Although there was a slight improvement, she still complained of persistent pain. In the upper part of the mucosa, 250 U of Dysport (Ipsen Biopharm Ltd., Slough, UK) was injected. As a transient side effect, food was spilled due to the unnatural movement of the lips at meal time, but the pain was significantly reduced. Three months later, the second injection of Dysport 250 U was performed. Thereafter, the pain was alleviated and the treatment was terminated.

### Case 2 (Table 1, no. 4)

A 52-year-old woman was referred to an oral and maxillofacial surgeon because of intermittent severe pain in the left facial area after facial nerve reconstruction during plastic surgery. During examination, the patient complained of general stiff pain in the facial region including the left zygoma, masseter, and temporomandibular joint area. There were also symptoms of excessive salivation in the left cheek. The patient was diagnosed with painful traumatic trigeminal neuropathy. Taking Trileptal did not improve the pain; therefore, it was replaced with Neurontin. At the same time, 25 U of Innotox (Medytox Inc., Seoul, Korea) was injected into the left masseter and physical therapy was performed. Physical therapy was performed five times with Sellalux (Medical United, Seoul, Korea), a low-level laser treatment unit, every 1 to 2 months. Two weeks after the first injection, the pain was significantly reduced. However, it persisted in the lower left eye and around the zygoma. Six months later, the second injection was performed with the same dose. Even though almost all the pain was alleviated, she still complained of pain in the stylomastoid foramen area under her left ear. Three months later, a third injection of 25 U of Innotox was performed and the pain was markedly reduced to a tolerable level.

All seven patients involved in this study were women with ages from 52 to 71 years. Five of the seven patients complained of pain in the oral mucosa, vestibule, teeth, and around the lips. Two of the patients complained of facial pain in the zygoma, masseter, and temporal muscle. The primary drugs used were carbamazepine, oxcarbazepine, gabapentin, pregabalin, amitriptyline, and nortriptyline. Physical therapy with either soft laser or EAST was performed additionally in most cases. Two of the patients were treated with Tegretol (carbamazepine), six were treated with Trileptal (oxcarbazepine), six were treated with Neurontin (gabapentin), two were treated with Lyrica (Pregabalin), one was treated with amitriptyline, and three were treated with Sensival (nortriptyline). Laser therapy was performed on all seven patients, while EAST was performed on two patients. Of the seven patients, five patients complained of oral pain. They received injections at the affected site. The remaining two patients received injections outside the oral cavity, specifically in the masseter and temporal muscles. Three types of BTX-A were used: Meditoxin (Medytox Inc., Seoul, Korea), Innotox, and Dysport (Ipsen Biopharm Ltd., Slough, UK), and the number of injections ranged from at least once to a maximum of three times every 3 months. In five of the patients with painful traumatic trigeminal neuropathy, pain was significantly reduced after BTX-A injection. The neuropathic pain in all patients who received implant surgery or facial nerve reconstruction was improved. However, in two patients with atypical orofacial pain who complained of persistent pain without any causative factors, the pain did not disappear after the injection. These patients were referred to the anesthesiology department for stellate ganglion block (SGB), but this treatment was not effective either (Table 1). After BTX-A injection, transient facial unbalance, unnatural movements, and facial asymmetry occurred. However, all these symptoms disappeared over time and there were no more complaints.

Traditionally, botulinum toxin has been used to treat strabismus and eyelid seizures. Since then, it has become widely used for the treatment of cervical dystonia and hyperhidrosis and for esthetic purposes [5]. Botulinum toxin is used to reduce masseter muscle volume for facial cosmetic purposes. Additionally, it is applied for the treatment of persistent myofascial pain and masticatory muscle disorders [6]. In addition, when unspecified temporomandibular joint pain and headache are reported, symptoms can be improved by injecting botulinum toxin into the masseter or temporal muscle. Botulinum toxins have been applied in many of these areas.

When unspecified facial pain persists, various treatments, ranging from conservative physical therapy and medication to surgical treatment, can be applied. Conservative treatments usually include medication, physical therapy, and splint therapy. Medication is primarily used to treat various facial pain such as unspecified temporomandibular joint disorders, neuropathic pain, atypical facial pain, and facial pain caused by rheumatoid arthritis. The drugs used include non-steroidal anti-inflammatory drugs (NSAIDs), analgesics, antidepressants, sedatives, muscle relaxants, and steroids. It is most important to diagnose the type and cause of facial pain and select the appropriate medication [7, 8]. Physiotherapy includes thermotherapy, cooling therapy, laser therapy, and electrical stimulation therapy. Of these, low-level laser therapy (LLLT) is the easiest to apply and produces no pain or discomfort. The laser has various effects such as improvement of vasodilation and blood circulation due to increase of temperature, relief of pain, relaxation of muscles, waste removal, and calming of the sensory nerve [9–11]. If conservative treatment fails, surgical treatment can be used, but the burden associated with invasive surgery and the risk of complications increase. In such cases, botulinum toxin can be a very useful therapy and has already proven its effectiveness in many studies. Freund et al. first reported treating temporomandibular disorder (TMD) with botulinum toxin [12, 13]. Since it has already been proven that parafunctions such as clenching and bruxism cause TMD, treatment with botulinum toxin was tested, and the effect was positive. Injecting botulinum toxin into the masticatory muscles not only reduces parafunctions but also relieves masticatory pain. The botulinum toxin inhibits the release of acetylcholine from all parasympathetic and cholinergic postganglionic sympathetic neurons. Therefore, it can be applied to smooth muscles and gland tissues. The analgesic effect of botulinum toxin is also caused by the inhibition of acetylcholine release and is directly associated with nociceptors. Botulinum toxin can influence sensitizing mediators, alter afferents derived from muscle spindles, cause physiological changes in reflex and synergistic movements, produce autonomic effects, and cause neuroplastic changes in the processing of afferent somatosensory activity. Therefore, botulinum toxin can be used effectively to treat pain syndrome because it can relax muscles [14]. Glenn et al. conducted a study among patients treated with botulinum toxin for complaints of orofacial pain of unknown origin. The patients complained of various orofacial pain such as trigeminal neuralgia, temporomandibular joint disorder, chronic tensional headache, and neuropathic pain. Their pain was significantly reduced after injection of botulinum toxin [15]. Thomas et al. conducted a meta-analysis study of botulinum toxin injections in patients with trigeminal neuralgia and postherpetic neuralgia. They concluded that botulinum toxin injection can be an alternative if there are no definitive medical solutions for pain [16].

In this study, the dose of BTX-A used varied from 20 to 200 U. These differences in injection dose are due to differences in the conversion ratio of BTX-A. An onabotulinum toxin-A (ONA) to abobotulinum toxin-A (ABO) conversion ratio is approximately 1:3 to 1:4. And a new botulinum toxin type A (NBoNT) to onabotulinum toxin-A (ONA) conversion ratio is approximately 1:1. Meditoxin and Innotox are included in new botulinum toxin type A (NBoNT). Dysport is included in abobotulinum toxin-A (ABO) [17, 18]. All patients who received BTX-A injection for non-odontogenic orofacial pain were females. They received various medications and physical therapy, but these did not improve their symptoms. They therefore received BTX-A injections. Among these patients, traumatic neuropathic pains resulting from postoperative complications such as implant surgery or facial nerve reconstruction tended to be reduced after BTX-A injection. However, atypical facial pain with unknown cause was not relieved. Although transient facial asymmetry or unbalance occurred as a complication after BTX-A injection, such complications were not a major problem because the patient was more interested in relieving the pain she was experiencing.

## Conclusions

Combining BTX-A injections with other treatments for traumatic trigeminal neuropathic pain that does not respond to conventional medication and physical therapies may be clinically useful. However, it is important to explain the complications of this treatment such as transient facial asymmetry to patients and to perform the treatment only after obtaining the patient’s consent.

## Additional file

Additional file 1: Case form and result of data. (XLSX 13 kb)

## Abbreviations

BTX-A: Botulinum toxin-A
EAST: Electric acupuncture stimulation therapy
LLLT: Low-level laser therapy
NSAIDs: Non-steroidal anti-inflammatory drugs
SGB: Stellate ganglion block
